# Inhibition of breast cancer cell growth by methyl pyropheophenylchlorin photodynamic therapy is mediated though endoplasmic reticulum stress‐induced autophagy in vitro and vivo

**DOI:** 10.1002/cam4.1418

**Published:** 2018-03-25

**Authors:** Jiang Zhu, Si Tian, Kai‐Ting Li, Qing Chen, Yuan Jiang, Hai‐Dan Lin, Le‐Hua Yu, Ding‐Qun Bai

**Affiliations:** ^1^ Department of Rehabilitation the First Affiliated Hospital of Chongqing Medical University Chongqing China; ^2^ Department of Rehabilitation Southwest University Hospital Chongqing China; ^3^ Department of Rehabilitation the Second Affiliated Hospital of Chongqing Medical University Chongqing China

**Keywords:** Autophagy, endoplasmic reticulum stress, methyl pyropheophenylchlorin, photodynamic therapy

## Abstract

Autophagy and ER stress participated in the inhibition of MPPa‐PDT on tumor growth, but the molecular links between them remain undefined. We just explore the molecular mechanism between them in vitro and vivo. CCK‐8 assay and flow cytometer were used to detect the cytotoxicity and mode of cell death after MPPa‐PDT. Furthermore, the role of autophagy was verified in MPPa‐PDT. Confocal microscopy was used to show the intracellular distribution of MPPa. ER stress markers and PERK signaling pathway were detected by western blot. While in vivo, tumor histology and immunohistochemistry were performed to show the effect of MPPa‐PDT in mice. After MPPa‐PDT, cells viability decreased in dose‐dependent manner. Besides, the cell apoptosis increased along with the increasing of Beclin‐1and LC3B II but declining of P62. When pretreated with 3‐MA, LC3B II formation and the cytotoxicity declined. MPPa‐PDT caused increasing of ER stress markers (GRP78, CHOP) as MPPa accumulated in ER. However, pretreatment with ER stress inhibitor 4PBA, the expression of GRP78 and LC3B II was blocked but the PERK signaling pathway activated and the expression of P62 increased. In vivo, the tumor growth was significantly inhibited by MPPa‐PDT. Besides, the appearance of ER stress and autophagy was further demonstrated by immunohistochemistry. Our findings demonstrate that autophagy mediated by MPPa‐PDT was regulated by ER stress, via PERK signaling pathway, to kill MDA‐MB‐231 cells in vitro and vivo.

## Introduction

Autophagy is a dynamic catabolic process where intracellular membrane structures including damaged cytoplasmic organelles and protein complexes are engulfed within membrane‐bound autophagosomes that fuse with lysosomes to transform autolysosomes, which then aim to degrade and renew their components 1, 2. Various physiological and pathological conditions trigger autophagy, such as nutrient deprivation, hypoxia, and drug exposure 3. As reported, the role of autophagy is complex, which can resist or promote cancer cell death under different stress conditions 4. When the autophagy process is activated by stress stimulate, the fate of cells is highly dependent on the cellular context and the strength and duration of the stress 5. During the last decade, there has been focused attention on the important role of autophagy in cancer research especially in anticancer therapy 6, 7, 8. Thus, a better understanding of the role autophagy takes and its mechanism in anticancer therapy would help us to devise more efficient therapies in the treatment of cancer.

The endoplasmic reticulum (ER) is a vital organelle required for Ca^2+^ homeostasis and proteins synthesis, folding, and modification 9. ER is also a highly sensitive organelle, and its complex functions can be significantly altered by a multitude of parameters including hypoxia, ER‐Ca^2+^ depletion, oxidative injury, and other factors, leading to ER stress. And then cells react to ER stress by activate a complex intracellular signal transduction pathway, known as the Unfolded Protein Response (UPR), which aimed at to reestablish ER homeostasis to adaption and safeguarding cellular survival or elimination of the damaged cell 10. Several studies have shown that severe ER stress via complex signal transduction pathway activates the autophagy, and furthermore, the autophagy can determine survival of cells concurrently with ER stress 11. Recently researches emphasized the importance of the ER stress and autophagy induced by anticancer therapies.

Although arising public awareness and advancement in medical science, breast cancer remains one of the leading causes of death in women 12. Less harmful, more effective and safe therapeutics are needed. Comparing to other breast cancer subtypes, triple‐negative breast cancer (TNBC) had limited choices of treatment. It is meaningful to find an effective treatment method.

Photodynamic therapy (PDT) is an anticancer therapy that relies on the selective photosensitizing of tumor cells, followed by visible light irradiation at the appropriate wavelength. The end result is formation of reactive oxygen species (ROS) that can cause photodamage to the cell 13, 14. Methyl pyropheophenylchlorin (MPPa), one of the derivatives, is a novel second‐generation photosensitizer and has been shown to be a potent photosensitizer. A number of studies have demonstrated that MPPa‐PDT can have a good anti‐cancer effect in most tumor cell lines 15, 16, 17.

The purpose of this study was to determine the photodamage function of MPPa–PDT in breast cancer cells and further investigate the effect of the interlink between autophagy and ER stress on the potential mechanism of MPPa‐PDT‐induced cell death in MDA‐MB‐231 cell in vitro and in vivo.

## Materials and Methods

### Major reagents

A stock solution of MPPa (Sigma‐Aldrich, St Louis, MA) was prepared by suspending it in a solution of DMSO at the concentration of 10 nmol/L and was sterilized by filtration with a 0.22‐*μ*m membrane (Millipore, Bedford, MA) stored at‐ 40°C. A stock solution of 3‐methyladenine (3‐MA) (Sigma‐Aldrich) and 4 phenylburyric acid (4PBA) (Sigma‐Aldrich) were prepared in PBS at the stock solution concentration of 50 mmol/L and 100 mmol/L, respectively. As required for individual experiments, the stock solutions were diluted by medium at the work concentration.

### Cell culture

The human breast cell line MDA‐MB‐231 (Shanghai Institute of cell biology China) was cultured in DMEM (HyClone, Logan, UT) supplemented with 10% fetal bovine serum and 1% penicillin and streptomycin (HyClone), and the cells were maintain in a humidified atmosphere containing 5% CO_2_ at 37°C.

### Photodynamic treatment

The MDA‐MB‐231 cells were randomly divided into four groups including control group (free drug and free light), single MPPa group, single light group, and MPPa‐PDT group. The cells of single MPPa group and MPPa‐PDT group were incubated with MPPa for 24 h, and then, the cells of MPPa‐PDT group and single light group were placed in the free drug medium followed by a red light‐emitting diode (LED) irradiation at the wavelength of 630 nm with a fluence rate of 30 mW/cm^2^ (total light dose (J/cm^2^) = fluence rate (mW/cm^2^) × treatment time (s).

### Cell viability assay

The MDA‐MB‐231 cells were seeded in 96‐well culture plates at a density of 1 × 10^4^ cells/well and incubated overnight at 37°C. The cells were divided into different treatment groups. At indicated time points, the viability of MDA‐MB‐231 cells was detected with the CCK‐8 assay (Beyotime, China) and absorbance of each sample was measured using a microplate reader (Tecan Group Ltd, Mannedorf, Switzerland) at 450 nm. Cell viability was calculated by percentage compared to the control group.

### Annexin V‐fluorescein isothiocyanate (FITC)/Propidium iodide (PI) staining

Flow cytometry was used to determine whether MDA‐MB‐231 cells treated with MPPa‐PDT underwent apoptosis or necrosis .Annexin V‐fluorescein isothiocyanate (FITC)/propidium iodide (PI) staining (Beyotime, China) was performed to detect early or late apoptosis. The MDA‐MB‐231 cells were seeded in 6‐well plates at 1 × 10^6^ cells per well and incubated with medium for a 24 h. Then the cells were divided into different treatment groups. At post‐treatment 24 h, the cells were harvested and centrifuged. The cells were incubated with Annexin V‐fluorescein isothiocyanate (FITC)/Propidium iodide (PI) for 5 min in the dark. The cell death was analyzed using flow cytometry (Becton Dickinson, San Joes, CA).

### Monodansylcadaverine staining

To assessment whether MDA‐MB‐231 cell treated with MPPa‐PDT induce autophagy, we observed autophagic vacuoles by monodansylcadaverine (MDC) staining. In 24‐well plates, MDA‐MB‐231 cells were seeded at a density of 5 × 10^3^ cells and cultured for 24 h. Then, the cells were treated with MPPa‐PDT and incubated for a 24 h. The cells were treated with 0.05 mmol/L MDC staining for 15 min and observed by fluorescence microscope (NIKON, Japan) and fluorspectrophotometer (Tecan Group Ltd) at indicated time.

### Western blot analysis

In order to observe the protein expressions level, MDA‐MB‐231cells were treated with PDT for 0–24 h and then were lysed in RIPA buffer (Beyotime, China) supplemented with protease inhibitors (PMSF) to extract total protein. And proteins were loaded on 15% (for light chain LC‐3, CHOP EIFA antibody), 8% (for PERK and GRP78/Bip antibody), or 10% (for P‐acting and atg12‐5 antibody) moeratuDS‐polyacrylamide gel and transferred to nitrocellulose membrane (Millipore). The blot was incubated in 5% nonfat milk for 2 h at room temperature and with the primary antibodies (Sigma‐Aldrich: LC3BB;.Cell Signaling Technologies: P62, PERK, eIF2a, ATG12‐5, CHOP, BIP and GAPDH) overnight at 4°C. The blot was then incubated with respective horseradish peroxidase‐conjugated secondary antibody(Cell Signaling Technologies) for 1 h at room temperature. The chemiluminescence reaction was detected by an ECL detection kit (Kaiji Bio Co., Nanjing, China)) using the LAS 3000 Luminoimage Analyzer.

### Clonogenic survival assay

To determine long‐term effect of inhibition, cells were cultured overnight in a 6‐well plate (1500 cells per well) and received different treatments. After 7 days of post‐treatment, the cells were fixed with 4% paraformaldehyde and stained with crystal violet (Sigma‐Aldrich) to observe and count cell colonies.

### Subcellular staining

For localization of photosensitizer (MPPa), at first the cells were incubated with MPPa for 24 h and then upload ER‐Tracker^™^ green (Invitrogen, Paisley, UK) at a final concentration of 500 nmol/L to label the endoplasmic reticulum (ER). Finally, the nuclei were stained with DAPI, and the stained cells were observed using a laser confocal microscope (TCS SP5; Leica).

### Xenograft model and PDT therapy

To investigate PDT therapy had anticancer function in vivo as well as in vitro, the athymic female nude mice (4 mice/group, 4–6 weeks old) were used for the tumor xenograft models. All animal experiments were approved by the Ethical Committee of Chongqing Medical University. The mice were purchased from the Experimental Animal Center of Chongqing Medical University [Certificate: SCXK (Yu) 2012–0001]. 1 × 10^6^ MDA‐MB‐231 cells in 100 *μ*L of PBS were injected subcutaneously into the back of mice (where it is convenient for light irradiation). When the diameter of tumor was about 0.5 ± 0.1 cm, nude mice were randomly divided into four groups including control group (free drug and free light), single MPPa group, single light group, and MPPa‐PDT group. MPPa was administered at a dose of 15 mg/kg 18 h before illumination with 120 J/cm^2^ light at 630 nm wavelength every other day for 10 days 16. Tumor measurement was performed for 30 days after PDT treatment. Tumor volume was measured every three days and calculated by the formula Tumor Volume (TV)  = Length × Width^2^/2.

### Tumor histology and Immunohistochemistry

After treatment with MPPa‐PDT, the tumor samples were removed and fixed with 4% paraformaldehyde for 2 days. The tumor samples were sectioned into five slices, and these were embedded in paraffin. Ten‐micrometer‐thick tumor sections were stained with hematoxylin–eosin reagent or set aside for IHC analysis .The sliders for IHC study were dewaxed, hydrated through graded alcohols and microwaved in 100 mmol/L sodium citrate for 5 min on high power and 10 min on low power for antigen retrieval. IHC analysis was performed following the protocol for the DakoCytomation EnVision + Dual Link System (Carpinteria, CA). Sections were incubated overnight at 4°C with rabbit polyclonal anti‐BIP and anti‐LC3B (1:100 dilution; Oxford Biomedical Research). BIP and LC3B positive cells were counted using MetaMorph Imaging Software (Universal Imaging Corporation; Downington, PA).

### Statistical analysis

The statistical analysis was conducted using SPSS 20.0 for Windows software. Data were present as mean ± SD in different experiments and were analyzed by one‐way analysis of variance (ANOVA) or two‐tailed Student's *t*‐test. A difference of *P *< 0.05 was considered statistically significant.

## Results

### PDT suppresses cell viability and induces cell apoptosis

**Figure 1:**
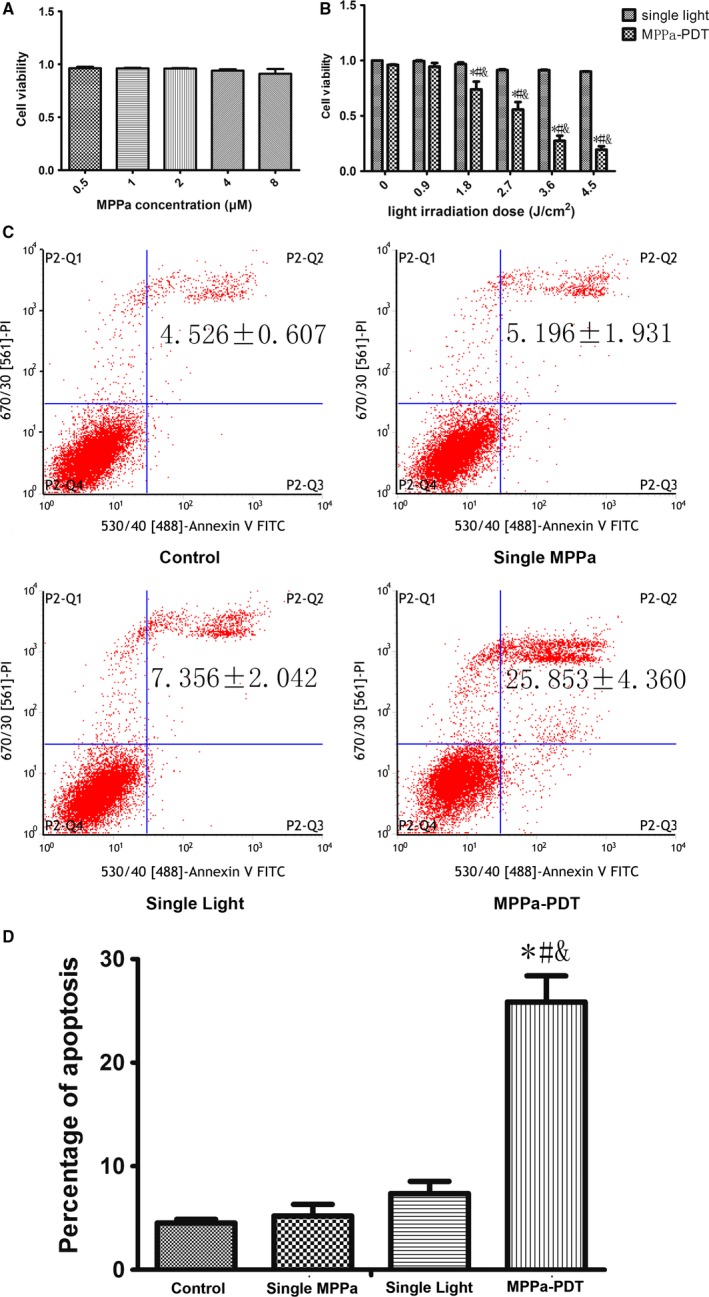
MPPa‐PDT produced cytotoxicity and apoptosis in MDA‐MB‐231 cells. (A) Cell viability of MPPa at different concentrations detected by the CCK‐8 assay. (A) Cell viability of MPPa at 2 *μ*M and different does of light detected by the CCK‐8 assay. (C) Apoptosis detected by annexin V/PI double‐staining. (D) Percentage of apoptosis. *P < 0.05, MPPa‐PDT vs. control. #*P* < 0.05, MPPa‐PDT vs. Single MPPa; &*P* < 0.05 MPPa‐PDT vs. Single light. Values are mean ± SD of three independent determinations.

Cells were incubated for 24 h with various concentrations of MPPa in dark for 24 h. Cell cytotoxicity was evaluated using CCK‐8 reduction assay. No significant dark cytotoxicity was observed at the dose range of 0.5–8 *μ*mol/L (Fig. 1A). So we chose the MPPa at the concentration of 2 *μ*mol/L base on results obtained in the literature 15, 18. Then, the cytotoxic effects of MPPa‐PDT were evaluated. After incubation with 2 *μ*mol/L MPPa for 24 h, the cells were irradiated by a series of light doses induced a decrease in cell viability in dose‐dependent manner (Fig. 1B). The median level of cytotoxicity of cells exposed to MPPa‐PDT was found at the light dose of 2.7 J/cm^2^ (Fig. 1B). So, we chose the concentration of MPPa at 2 *μ*mol/L and light dose at 2.7 J/cm^2^ for the subsequent experiments. The results of flow cytometry shown that the apoptotic rate of LC50 (2 *μ*mol/L + 2.7 J/cm^2^) was 25.853 ± 4.36% in MPPa‐PDT group, which was significant higher than other three groups (*P *<* *0.05), However, these was no differences among the other three groups (*P *>* *0.05) (Fig. 1C,D).

### PDT induces autophagy

**Figure 2:**
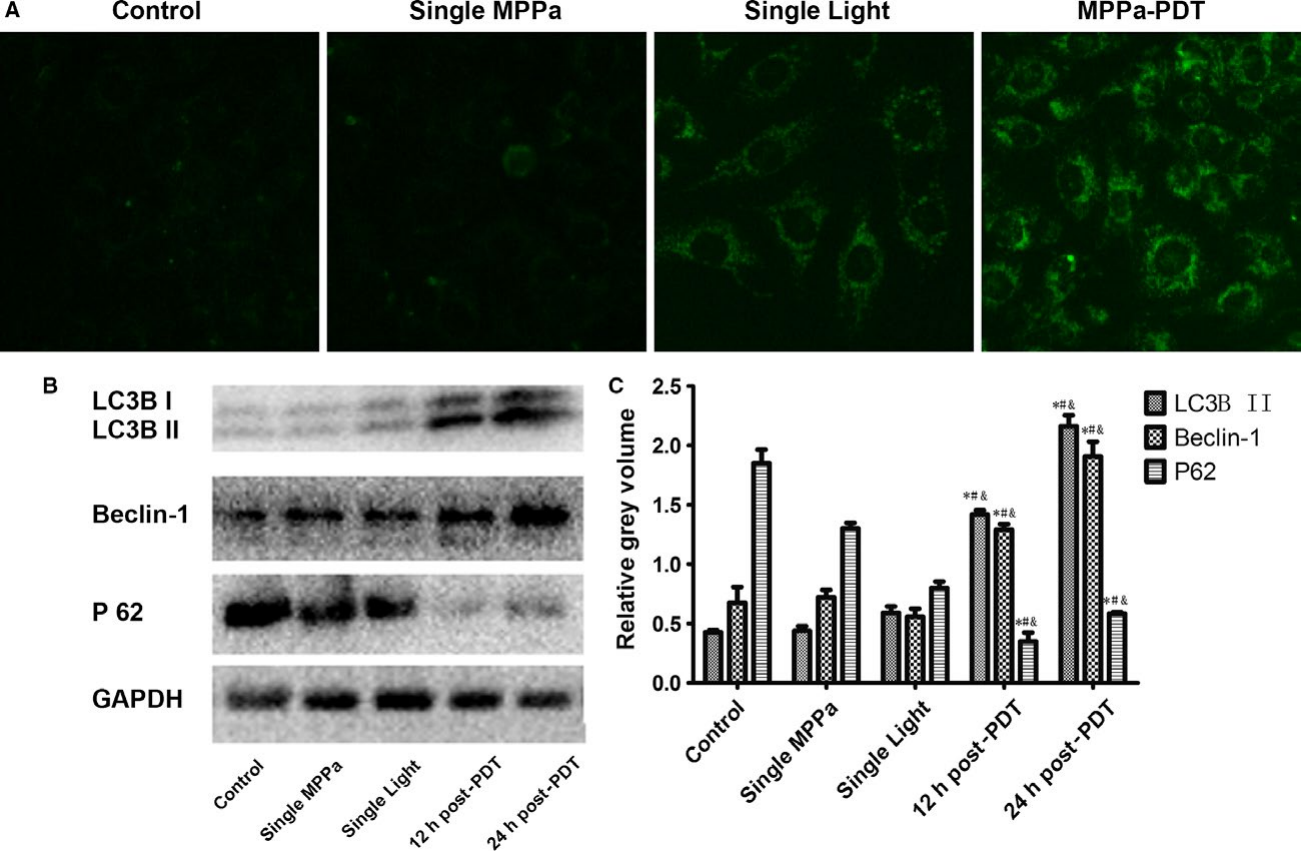
MPPa‐PDT induces autophagy. (A) The expression of autophagic vacuoles detected by immunofluorescence was observed by monodansylcadaverine (MDC) staining (MDC,×200). MDC (green) staining. (B) Expression of LC3B II, Beclin‐1, P 62 proteins Lanes; (C) Relative Gray volume. *P < 0.05, MPPa‐PDT vs. control. #*P* < 0.05, MPPa‐PDT vs. Single MPPa; &*P* < 0.05 MPPa‐PDT vs. Single light. Values are mean ± SD of three independent determinations.

As shown in Figure 2A, it is suggested that MPPa‐PDT caused a relatively large increase in positive MDC fluorescent staining, visualized as a green fluorescent signal. Western blot analysis showed that when exposure to MPPa‐PDT, the protein expression of LC3 B and Beclin‐1 was significantly increased; however, the protein expression of P62 was significantly declined when compared to cells in control groups (Fig. 2B,C). These data suggest that autophagy was induced during MPPa‐PDT in MDA‐MB‐231 cells.

### Autophagy plays a role in the death of MDA‐MB‐231 cells treated with PDT

**Figure 3:**
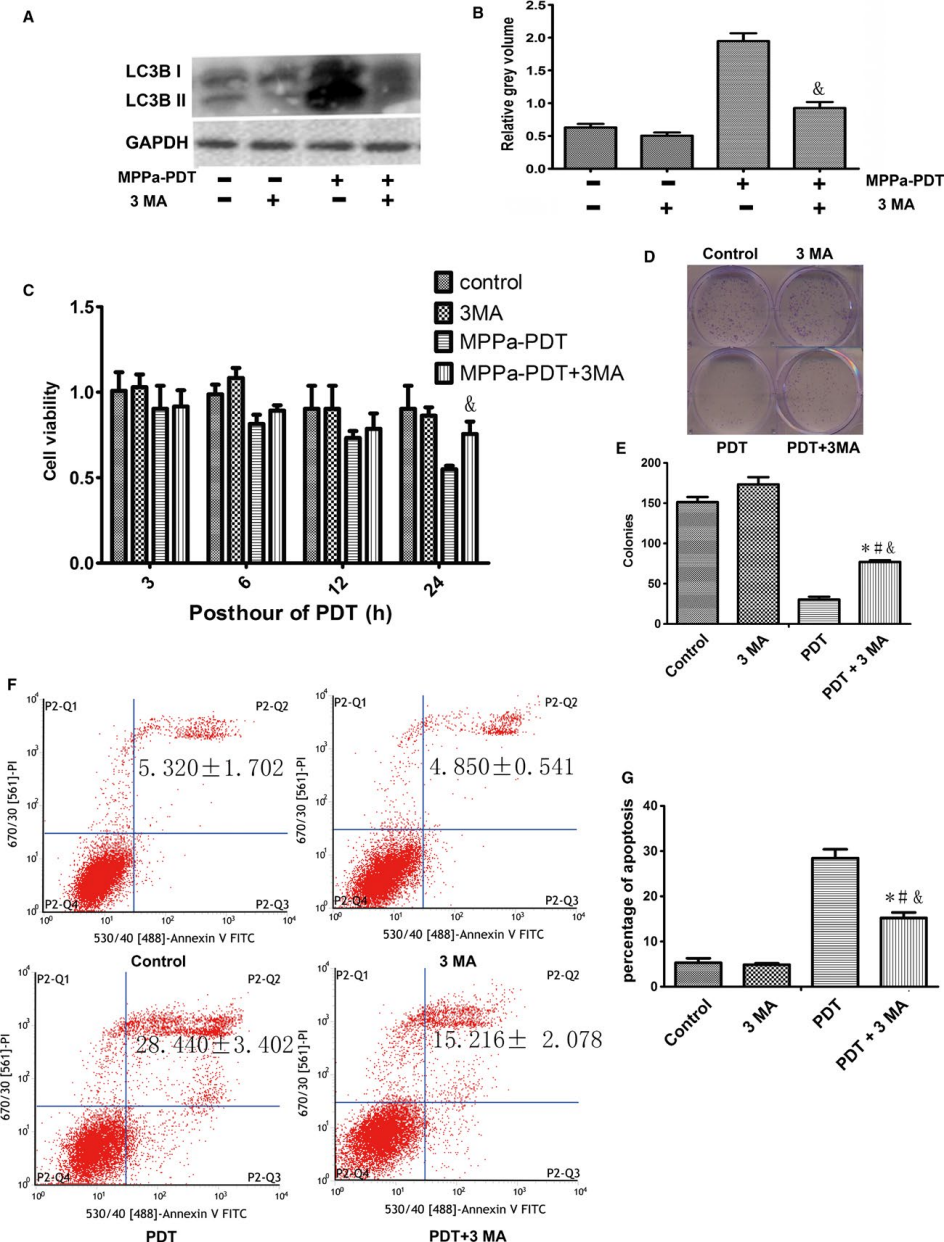
Effects of 3‐MA on MPPa‐PDT induced cell death. (A) Expression of LC3B II proteins Lanes; (B) Relative Grey volume of LC3B II; (C) Cell viability of MPPa‐PDT + 3 MA at different hours post PDT, detected by the CCK‐8 assay; (D) The long proliferation was detected by colony‐forming assay staining with crystal violet; (E) The number of colony‐forming unit; (F) Apoptosis detected by annexin V/PI double‐staining; (G) Percentage of apoptosis *P < 0.05, MPPa‐PDT + 3MA vs. control. #*P* < 0.05, MPPa‐PDT+3MA vs. 3 MA; &*P* < 0.05 MPPa‐PDT + 3MA vs. MPPa‐PDT. Values are mean ± SD of three independent determinations.

As shown in Figure 3, the MDA‐MB‐231 cells pretreated with 5 mmol/L 3‐MA for 1 h prior to PDT had a decrease expression in LC3B II protein level and a significantly decline in cytotoxicity and apoptosis, but with an increase in colonies formation when compared to cells treated with PDT alone (Fig. 3). In summary, autophagy can be playing a role as a death mechanism in MPPa‐PDT in MDA‐MB‐231 cells.

### PDT induces ER Stress

**Figure 4:**
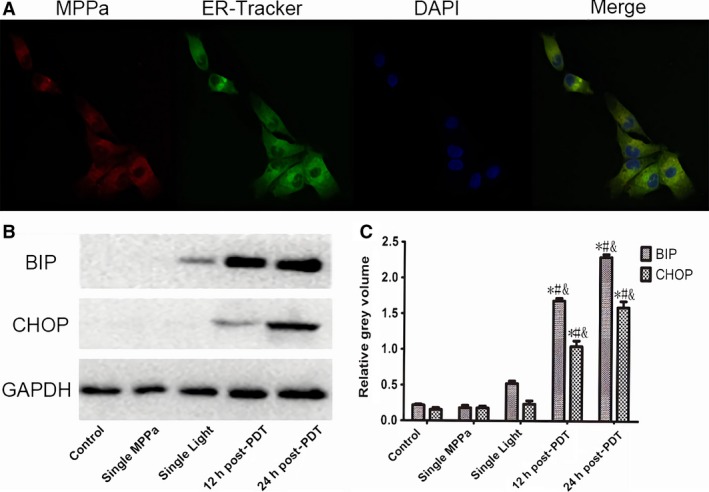
PDT induces ER Stress. (A) The location of MPPa detected by confocal microscopy was observed by ER‐Tracker^™^ green staining (×400). (B) Expression of BIP, CHOP proteins Lanes; (C) Relative gray volume; *P < 0.05, MPPa‐PDT vs. Control. #*P* < 0.05, MPPa‐PDT, vs. Single MPPa; &*P* < 0.05 MPPa‐PDT vs. Single light. Values are mean ± SS.D. of three independent determinations.

As shown in Figure 4, MPPa entered into MDA‐MB‐231 cells and was localized to the cytoplasm around the nucleus. The red fluorescent signal of MPPa overlapped with the green fluorescent signal of ER suggested that MPPa accumulated in ER of the cells after 24‐h incubation. And the study shows that treatment with PDT induced a time‐dependent increase in the protein expression levels of ER stress markers BIP and CHOP in MDA‐MB‐231 cells (Fig. 4B,C).

### The PERK/eIF2a Signaling Pathway Is Required for PDT induced Autophagy in MDA‐MB‐231 cells

**Figure 5:**
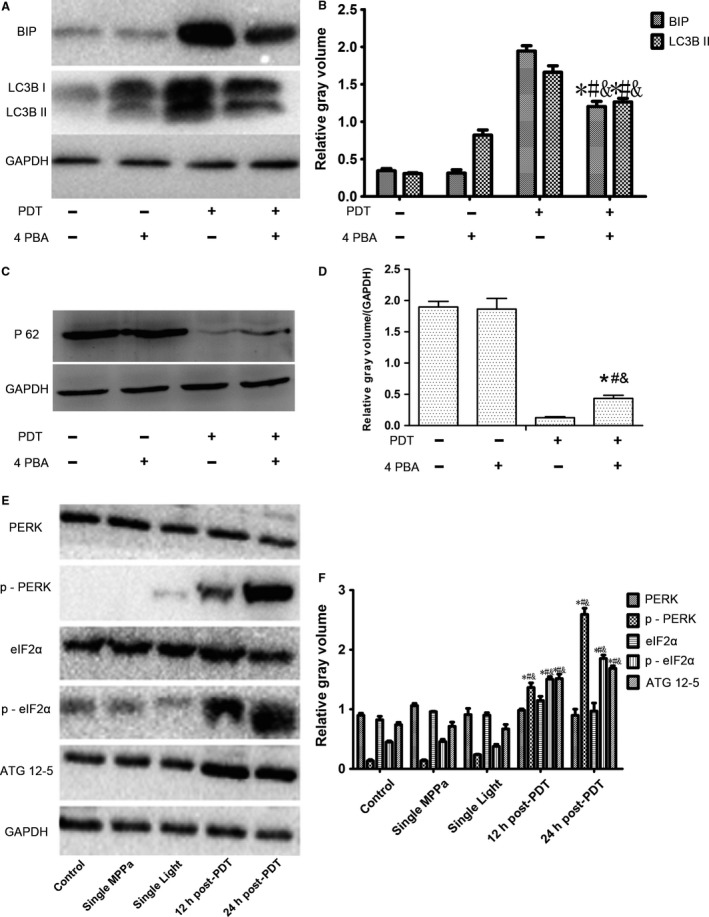
The relationship between autophagy and ER stress. (A) Expression of BIP and LC3B proteins Lanes; (B) Relative gray volume of BIP and LC3B II; (C) Expression of P 62 proteins Lanes; (D) Relative Grey volume of P 62; *P < 0.05, MPPa‐PDT + 4PBA vs. Control. #*P* < 0.05, MPPa‐PDT + 4PBA vs. 4PBA. &*P* < 0.05 MPPa‐PDT + 4PBA vs. MPPa‐PDT. (E) Expression of PERK/eIF2a Signaling Pathway protein Lanes; (F) Relative gray volume. *P < 0.05, MPPa‐PDT vs. Control. #*P* < 0.05, MPPa‐PDT vs. Single MPPa; &*P* < 0.05 MPPa‐PDT vs. Single light. Values are mean ± SD of three independent determinations.

When MDA‐MB‐231 cells pretreated with 10 mmol/L 4PBA for 12 h before exposed to PDT, there was a large decrease in the protein expression levels of LC3B and BIP when compared to cells in control groups, but increase of P62 (Fig. 5A–D). It also showed that treatment with PDT induced an increase in the protein expression levels of PERK/eIF2a signaling pathway proteins including p‐PERK, p‐eIF2*α*, ATG12‐5 (Fig. 5E,F).This results demonstrates that ER stress plays an important role in regulating autophagy in PDT treatment .

### PDT inhibits growth of MDA‐MB‐231 cells

**Figure 6:**
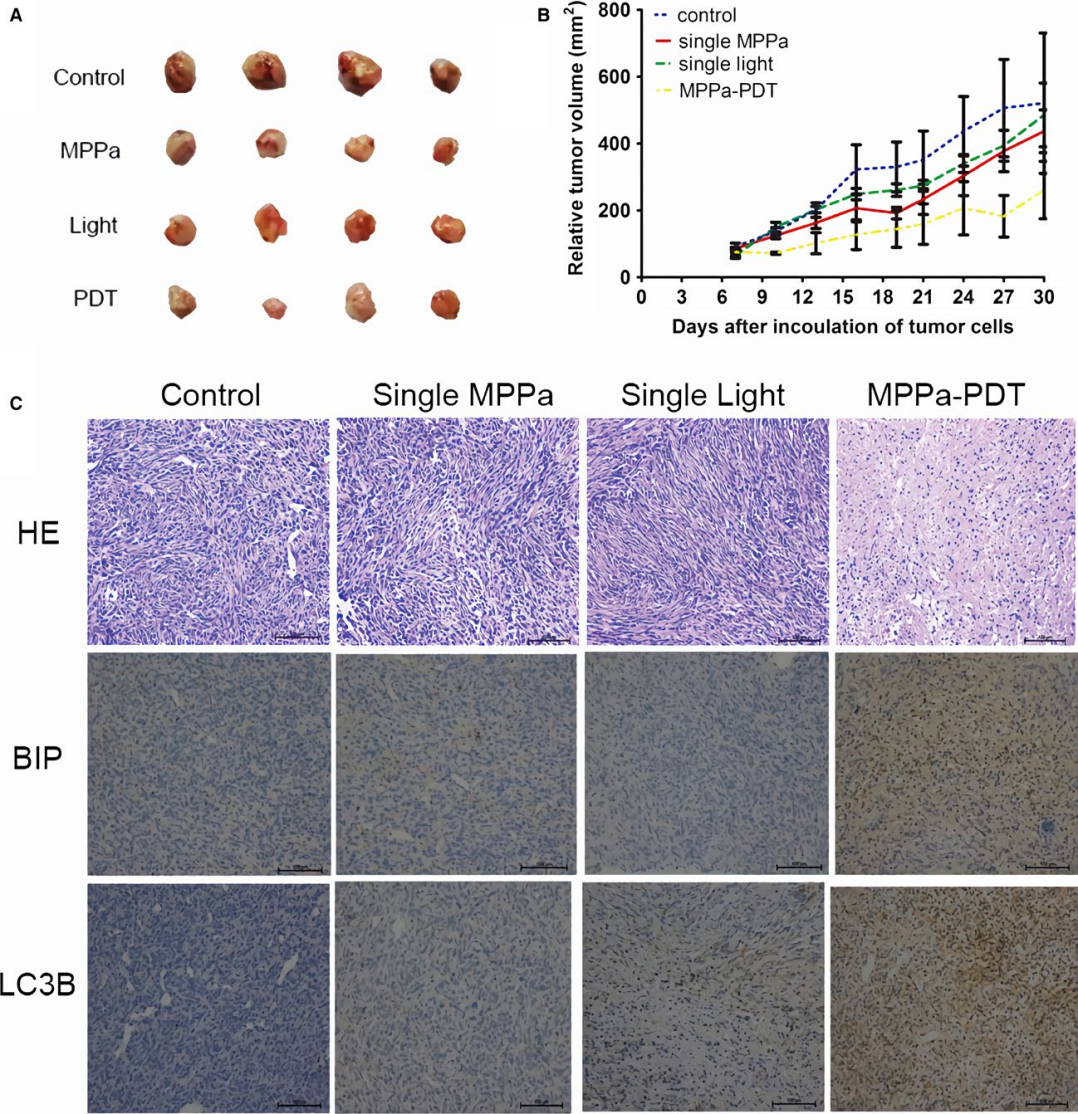
PDT inhibits growth of MDA‐MB‐231 cells. (A) Athymic mice were implanted with MDA‐MB‐231 tumors and mice (*n *= 4) were treated with MPPa (15 mg/kg), light (120 J/cm^2^), MPPa‐PDT as described in the materials and methods. At point time, tumors were excised. (B) Tumor volume was measured every 3 day after the diameter of tumor was about 0.5 ± 0.1 cm (about the seventh day). (C) Tumor samples histology was detected by HE staining. Expression of BIP and LC3B proteins was detected by ICH.

In this present study, it suggested that PDT activated the ER stress autophagy signal pathway in MDA‐MB‐231 cell lines in vitro. To further evaluate these findings in vivo, we explored the antitumor effect of PDT in mice harboring MDA‐MB‐231 cell lines xenografts. Mice were treated with MPPa alone, light irradiation alone and both agents together, respectively. MPPa‐PDT suppressed tumor volume by 2 times compared with control group (Fig. 6A,B). And many necrosis cells could be seen in MPPa‐PDT group by histopathological examinations (Fig. 6C). The proteins of LC3B and BIP were significantly expressed in MPPa‐PDT group (Fig. 6C). These results suggest that PDT suppresses the growth of MDA‐MB‐231 human breast cancer cell in vivo.

## Discussion

PDT, an anticancer method with minimal toxicity to normal tissues, is effective in most tumor cells 19, 20, 21. Apoptosis is a major type of programmed cell death utilized in anticancer treatment. A research demonstrated that PDT could lead to the initiation of the apoptotic death processes 14. This study demonstrated the effect of MPPa‐PDT in human MDA‐MB‐231 breast cancer cells in vitro .The results showed that PDT can inhibit the growth of the MDA‐MB‐231 breast cancer cell. And then apoptotic rate analysis also proved the effectiveness of PDT.

Interestingly, the apoptotic rate (25.853 ± 4.36%) was significantly lower compared to the cytotoxicity rate (44.306 ± 11.779%) in MPPa‐PDT group detected by CCK‐8 assay, so we speculate that there may be other pathways leading to the cell death. As a death mechanism implicated in cancer research 22, autophagy was investigated to uncover its role in MPPa‐PDT in MDA‐MB‐231 cells. Standard test for autophagy include MDC staining and the expression of microtubule‐associated protein 1 light chain 3 (LC3) and BECLIN 1 23, 24. Accumulation of autofluorescent monodansylcadaverine (MDC) in mature autophagic vacuoles (autolysosomes) is an important marker for autophagy, and it is directly related to induction of autophagy 25, 26. The conversion of LC3B‐I to LC3B‐II has been thought to be a vital role in elongation of the isolation membrane and eventual closure of the autophagosomal membrane, so it is used as a marker for monitoring the autophagic process 23. The other autophagy protein, Beclin 1, is a central regulator of autophagy and functions through its interaction with the Class III PI3K VPS34, VPS15 to regulate autophagy process 24. Our data showed that treatment with a cytotoxic dose of PDT caused a decrease in autophagy markers (Fig. 2). The final outcome of activation of the autophagy program is highly dependent on the cellular context and the stress signals. Furthermore, our study showed that cells pretreated with 5 mmol/L 3‐MA, an inhibitor of autophagy, which blocked the conversion of LC3B‐I to LC3B‐II, and thus, it revealed a reduction cytotoxicity of MPPa‐PDT in short and long observation period by CCK‐8 experiment and Clonogenic survival assay and a lower apoptosis rate compared to cells with MPPa‐PDT alone (Fig. 3). These findings indicated autophagy played an important role in anticancer effect via enhancing apoptosis to cause cells death induced by MPPa‐PDT in MDA‐MB‐231 cells.

Autophagy as a catabolic process is induced by stress to regulate cellular homeostasis 2. Different situations induce autophagy also can induce ER stress. Furthermore, the activation of ER stress leads to autophagy 27. To understand the mechanisms by which ER stress and autophagy are interconnected under PDT treatment, further studies were conducted to assess the ER stress level with PDT treatment. In PDT, the precise subcellular localization of the photosensitizer is attributed to the oxidative damage in cellular compartments including mitochondria, endoplasmic reticulum (ER), lysosomes, and Golgi apparatus 28, 29. Recent research has identified ER is a crucial regulator of cell death 30, 31. Our study demonstrated that MPPa can selectively accumulate in ER (Fig. 4). When ER is stimulated, there are a large number of regulatory components involved to cope with the change of microenvironment in ER. Among the many factors, the chaperone GRP78 (BIP) and CHOP are two key elements that stand out in stress stimulation. GRP78 (BIP) as an initiator of early ER stress/UPR signaling is influenced by the cellular and micro‐environmental disturbances, and it can activate and elevate along with aggravated ER stress 32. Indeed, GRP78 (BIP) has become an indicator and marker for the ER stresses 32. CHOP (C/EBP homologous protein) is a pro‐apoptotic factor that is kept very low in nonstressed cells but also not conspicuously expressed in tumor tissues or tumor cell line. But aggravated ER stress, CHOP expression is strongly stimulated through IRE1‐ and PERK‐mediated signaling and regulated the downstream transcription factors to carry out the apoptotic program 33, 34. There were consistent with the present findings in increase of BIP and CHOP in our study to demonstrated ER stress induced by MPPa‐PDT.

This study has demonstrated that PDT‐induced cytotoxicity was associated with the concurrent induction of autophagy and ER stress. Moreover, the lever of BIP was blocked with ER stress inhibitor 4PBA and accompanied by a reduction in autophagy (Fig. 5). Our findings showed that there is a connection between autophagy and ER stress. When ER stress is activated, the cells react to ER stress by activation of a complex intracellular signal transduction pathway named the unfolded protein response (UPR). UPR in mammalian cells is governed by three transmembrane ER stress sensors, namely PKR‐like ER kinase (PERK), inositol requiring enzyme 1 (IRE1), and activating transcription factor 6 (ATF6). Those transmembrane ER stress sensors are kept in an inactive state by binding GRP78 in nonstressed cells. When ER homeostasis is disrupted, accumulating misfolded proteins make more burden on ER function and UPR is activated by depart away BIP to regulated downstream transcription factors 31, 35, 36. In these three sensors, PERK single pathway of ER stress plays an important role in the activation of autophagy in response to UPR 37, 38. PERK is a type I transmembrane protein with a luminal sensing domain and a cytosolic kinase domain that cause transautophosphorylation by activating downstream transcription factors eukaryotic initiation factor 2 alpha (eIF2*α*) 37, 39, 40. Some studies show that when cells are exposed to a negative form of PERK or genetic substitution of Serine of eIF2*α*, it can block formation of autophagy 41, which strongly suggests that PERK depends the phosphorylation of eIF2*α* to activate autophagy. It is still not clear how the signal pathways by which eIF2*α* phosphorylation can modulate autophagy work. But previous research has shown that the phosphorylation process of eIF2*α* regulates transcription factor 4 (ATF4), a transcription factor that stimulates a set of genes involved in supporting recovery and adaptation, causing the conjugation of LC3B I to Atg5‐Atg12 42, 43. Our study suggests that ER stress induced by MPPa‐PDT had been shown to be involved in activating the PERK‐eIF2*α* pathway and stimulating autophagy in MDA‐MB‐231 cells.

In vivo studies, the PDT effect on tumor was similar to that in vitro. A difference between treatment and control groups in tumor size was observed. Interestingly, MPPa‐PDT also induced necrosis along with autophagy and ER stress in vivo. Necrosis causing more cell death program inside of tumor was due to hypoxia and metabolic stress 44. These results suggest that the anticancer effect of PDT treatment was associated with multiple vital factors such as ER stress, autophagy, and necrosis in vivo.

In conclusion, we demonstrated that autophagy could represent a promising target in cancer treatment and ER stress may be a therapeutic regulator to influence autophagy to enhance the anticancer effect of PDT in human breast cancer cell. There is a good theoretical basis for the application of MPPa‐PDT in clinical setting.

## Conflict of Interest

The author(s) confirm that this article content has no conflict of interest.
